# Supplementary material for: Filter inference: A scalable nonlinear mixed effects inference approach for snapshot time series data
Source: PLoS Comput Biol. 2023 May 22;19(5):e1011135. doi: 10.1371/journal.pcbi.1011135 (PMC10237648; doi:10.1371/journal.pcbi.1011135)
Supplement: S1 Text — (PDF) [file pcbi.1011135.s001.pdf]

S1 Text. Optimised implementation of filter inference.

The implementation of filter inference can be optimised such that it solves the time series model only once for each simulated individual by leveraging the fact that numerical differential equation solvers can efficiently evaluate a system of differential at any number of time points. The optimised implementation, therefore, solves the time series model for each individual only once up front, computing $\bar{y}(\psi_s, t)$ for all measured time points in one time series model solve. This removes the unnecessary costs of integrating the time series model for each measured time point separately.
