# Supplementary material for: Filter inference: A scalable nonlinear mixed effects inference approach for snapshot time series data
Source: PLoS Comput Biol. 2023 May 22;19(5):e1011135. doi: 10.1371/journal.pcbi.1011135 (PMC10237648; doi:10.1371/journal.pcbi.1011135)
Supplement: S3 Text — (PDF) [file pcbi.1011135.s003.pdf]

S3 Text. Estimation of variance estimation error.

We estimate the variance at time point t_j using

$$\hat{\sigma}_j^2 = \frac{1}{n_j - 1} \sum_{i=1}^{n_j} \left(y_{ij} - \hat{\mu}_j\right)^2 ,$$

where n_j denotes the number of measurements at t_j and $\hat{\mu}_j = \sum_i y_{ij}/n_j$ denotes their mean. Assuming that y_{ij} are drawn from a Gaussian distribution $\mathcal{N}(y|\mu_j, \sigma_j^2)$ with mean μ_j and variance σ_j^2, the normalised variance estimator follows a χ^2-distribution with $n_j - 1$ degrees of freedom

$$\frac{n_j - 1}{\sigma_j^2} \hat{\sigma}_j^2 \sim \chi^2_{n_j - 1}.$$

The variance of a $\chi^2_{n_j-1}$ random variable is $2(n_j - 1)$. As a result, we can estimate the standard deviation of $\hat{\sigma}_j^2$ using

$$\sqrt{\mathrm{Var}\left[\hat{\sigma}_j^2\right]} = \sigma_j^2 \sqrt{\frac{2}{n_j - 1}} \approx \hat{\sigma}_j^2 \sqrt{\frac{2}{n_j - 1}}.$$

At $t = 0$, the measurement distribution in Fig 3A happens to be Gaussian, and the above estimate is unbiased.
