# Supplementary material for: Filter inference: A scalable nonlinear mixed effects inference approach for snapshot time series data
Source: PLoS Comput Biol. 2023 May 22;19(5):e1011135. doi: 10.1371/journal.pcbi.1011135 (PMC10237648; doi:10.1371/journal.pcbi.1011135)
Supplement: S4 Text — (PDF) [file pcbi.1011135.s004.pdf]

S4 Text. IIV-noise distinguishability.

To understand the IIV-noise distinguishability when estimating parameters from snapshot measurements using NLME inference and filter inference, we generate snapshot datasets from the early cancer growth model analogously to Early cancer growth model inference, but with increased numbers of measured individuals per time point. In particular, we generate datasets with $N = 90$, $N = 270$, $N = 810$ and $N = 2430$ individuals and estimate the parameters using NLME inference and filter inference with a Gaussian filter and $S = 100$ simulated individuals. The inference results are depicted in S1 Fig.

The figure shows that the uncertainty of the population mean estimates and the population standard deviation estimates decreases as the number of measured individuals increases, demonstrating that the estimation of IIV becomes more accurate as more individuals are measured. However, it also shows that for most of the datasets the posterior distributions of the noise parameter, μ_σ, do not substantially differ from the prior distribution. As argued in Early cancer growth model inference, this is because the prior distribution, $p(\mu_\sigma)$, focuses on values that give rise to noise magnitudes that are all compatible with the variability of the measurements, such that variability contributions from IIV and noise can only be distinguished if they give rise to different shapes of the measurement distribution, $p(y|\theta, t)$, and sufficiently many measurements are available to resolve such distributional differences. S2 Fig shows the measurement distribution for different samples from the posterior distribution in the top row of S1 Fig, demonstrating that the shape of the distribution does change for different contributions from IIV and noise, while the variance of the distribution remains the same.

Such distributional differences cannot be resolved by $N = 90$, $N = 270$ and $N = 810$ snapshot measurements, as demonstrated by columns 1-3 in S1 Fig. However, using snapshot measurements from $N = 2430$, NLME inference begins to have sufficient statistical power to resolve differences between IIV and noise, resulting in an update of $p(\mu_\sigma|\mathcal{D})$ relative to the prior (see bottom right corner of S1 Fig). Filter inference with a Gaussian filter and $S = 100$ simulated individuals is not able to distinguish IIV and noise, even when $N = 2430$ individuals are measured. This is a consequence of the filter approximation of the measurement distribution which only uses simulations of $S = 100$ individuals. While $N = 2430$ measurements start to resolve distributional

differences between IIV and noise contributions, $S = 100$ simulated individuals are insufficient to do so. Increasing the number of simulated individuals alleviates this limitation, as demonstrated in S3 Fig, where we illustrate posterior distributions inferred from $N = 60\,000$ snapshot measurements using filter inference with Gaussian filters and $S = 5\,000$ simulated individuals.
