# Supplementary material for: Filter inference: A scalable nonlinear mixed effects inference approach for snapshot time series data
Source: PLoS Comput Biol. 2023 May 22;19(5):e1011135. doi: 10.1371/journal.pcbi.1011135 (PMC10237648; doi:10.1371/journal.pcbi.1011135)
Supplement: S5 Text — (PDF) [file pcbi.1011135.s005.pdf]

S5 Text. Estimation of posterior average of population distribution.

Formally, the average of the population distribution over the posterior distribution, also known as the posterior predictive population distribution, is defined as

$$p(\psi|\mathcal{D}) = \mathbb{E}_{\theta|\mathcal{D}}[p(\psi|\theta)] = \int \mathrm{d}\theta\, p(\psi|\theta)\, p(\theta|\mathcal{D}).$$

While the integral is generally difficult, or impossible, to compute analytically, the above equation implicitly defines a joint distribution of the individual-level parameters and the population-level parameters, $p(\psi, \theta|\mathcal{D}) = p(\psi|\theta)\, p(\theta|\mathcal{D})$. To sample from $p(\psi|\mathcal{D})$, we can sample from $p(\psi, \theta|\mathcal{D})$ and marginalise over the sampled population-level parameters afterwards. In particular, we first sample a θ from $p(\theta|\mathcal{D})$, and then draw a ψ from $p(\psi|\theta)$ using the sampled θ. The histogram over the sampled ψ converges to the posterior predictive population distribution as the number of samples tends to infinity. In practice, samples from $p(\theta|\mathcal{D})$ are generated by sampling with replacement from the MCMC posterior samples.
