# Supplementary material for: Filter inference: A scalable nonlinear mixed effects inference approach for snapshot time series data
Source: PLoS Comput Biol. 2023 May 22;19(5):e1011135. doi: 10.1371/journal.pcbi.1011135 (PMC10237648; doi:10.1371/journal.pcbi.1011135)
Supplement: S6 Text — (PDF) [file pcbi.1011135.s006.pdf]

S6 Text. Estimation of EGF pathway model parameters.

The parameters of the EGF pathway model are estimated with pints' implementation of NUTS, unless otherwise specified. MCMC chains are run for 1500 iterations, where the first 500 iterations are used for calibration. The convergence is assessed using the $\hat{R}$-statistic from 3 MCMC chains, initialised at randomly sampled points from the prior distribution. The prior distribution for all inference runs is

$$\begin{aligned}
\mu_p &\sim \mathcal{N}(2, 0.25) \\
\sigma_p &\sim \mathrm{LN}(-2, 0.5) \\
\mu_{k_{\mathrm{on}}} &\sim \mathcal{N}(2, 0.25) \\
\sigma_{k_{\mathrm{on}}} &\sim \mathrm{LN}(-2, 0.5) \\
\mu_{k_{\mathrm{off}}} &\sim \mathcal{N}(10, 4) \\
\mu_{k_{\mathrm{deg},r}} &\sim \mathcal{N}(0.3, 0.0025) \\
\mu_{k_{\mathrm{deg},a}} &\sim \mathcal{N}(0.02, 0.000025).
\end{aligned}$$

Detailed information on the convergence can be found in S2 Table.
