# Supplementary material for: Filter inference: A scalable nonlinear mixed effects inference approach for snapshot time series data
Source: PLoS Comput Biol. 2023 May 22;19(5):e1011135. doi: 10.1371/journal.pcbi.1011135 (PMC10237648; doi:10.1371/journal.pcbi.1011135)
Supplement: S7 Text — (PDF) [file pcbi.1011135.s007.pdf]

S7 Text. Evaluation time estimation.

The evaluation times of the NLME log-posterior and the filter log-posterior in Fig 7 are estimated by evaluating the log-posteriors for each dataset 10 times at fixed parameter values on a MacBook Pro, 2.8 GHz Quad-Core Intel Core i7 processor with 16 GB 2133 MHz LPDDR3 memory. The execution time of each evaluation is measured using the Python package `timeit`. The minimum execution time of the 10 repeats is reported in the figure. In this way, we are able to find the optimal execution time in Python, accounting for Python's unpredictable overhead of execution time.
