# Supplementary material for: Filter inference: A scalable nonlinear mixed effects inference approach for snapshot time series data
Source: PLoS Comput Biol. 2023 May 22;19(5):e1011135. doi: 10.1371/journal.pcbi.1011135 (PMC10237648; doi:10.1371/journal.pcbi.1011135)
Supplement: S8 Text — (PDF) [file pcbi.1011135.s008.pdf]

S8 Text. Hyperparameter tuning of MH algorithm.

The hyperparameter of the MH algorithm is the step size of the proposal step. More precisely the MH algorithm proposes new parameters by sampling from a multivariate Gaussian distribution centred at the last accepted proposal. The covariance matrix of this distribution sets the scale of the step size and is the hyperparameter of the algorithm. To find a good set of step sizes we performed a grid search over several step sizes (see S4 Table). In particular, we used the variances of the filter posterior distributions inferred using NUTS in S1 Fig and Fig 6B as a starting point – information one would usually not have available prior to the inference. This allowed us to carry out the grid search more efficiently, as the marginal variances of the target distribution provide a good starting point for the diagonal of the covariance matrix of the MH proposal distribution. For each problem we ran the MH algorithm 7 times with 3 chains for 5000 MCMC iterations with diagonal covariance matrices whose diagonal was set to x times the variances of the target distribution. We performed the grid search for $x \in \{1.4, 1.2, 1, 0.8, 0.6, 0.4, 0.2\}$. The covariance matrix with the largest ESS was chosen across for 3 for the inference. All chains where randomly initialised in the vicinity of the data-generating parameters and the first 2500 iterations were discarded before the ESS computation.
