# Supplementary material for: Filter inference: A scalable nonlinear mixed effects inference approach for snapshot time series data
Source: PLoS Comput Biol. 2023 May 22;19(5):e1011135. doi: 10.1371/journal.pcbi.1011135 (PMC10237648; doi:10.1371/journal.pcbi.1011135)
Supplement: S9 Text — (PDF) [file pcbi.1011135.s009.pdf]

S9 Text. Equivalence of filter inference with a Gaussian filter and ABC based on the mean and variance.

Let $Y_1, \dots Y_N$ be N i.i.d. random, real-valued variables drawn from the data-generating distribution $q(y)$. Let q have nonzero variance, $\mathrm{Var}[Y] > 0$. Let $\tilde{Y}_1, \dots \tilde{Y}_S$ be S i.i.d. random variables drawn from the model $p(y|\theta)$. Let further $\mathcal{K}_\varepsilon(X - \tilde{X})$ be a kernel with an error margin ε, used in ABC to quantify the distance between the data summary statistic $X = X(Y_1, \dots Y_N)$ and the simulated summary statistic $\tilde{X} = X(\tilde{Y}_1, \dots \tilde{Y}_S)$. Let the kernel converge to a Dirac delta distribution up to a proportionality factor as the error margin goes to zero, $\lim_{\varepsilon \to 0} \mathcal{K}_\varepsilon(X - \tilde{X}) \propto \delta(X - \tilde{X})$. Let further $p(y|\tilde{\mu}, \tilde{\sigma}^2) = \mathrm{e}^{-(y-\tilde{\mu})^2/2\tilde{\sigma}^2}/\sqrt{2\pi\tilde{\sigma}^2}$ denote a Gaussian filter and $p(\mathcal{D}|\tilde{\mu}, \tilde{\sigma}^2) = \prod_{i=1}^N p(Y_i|\tilde{\mu}, \tilde{\sigma}^2)$ denote its likelihood, where $\mu = \sum_{i=1}^N Y_i/N$ denotes the mean estimator and $\sigma^2 = \sum_{i=1}^N (Y_i - \mu)^2/(N-1)$ denotes the variance estimator. Then, a Gaussian filter converges to a Dirac delta distribution between (μ, σ^2) and $(\tilde{\mu}, \tilde{\sigma}^2)$ up to a proportionality factor as the number of measurements goes to infinity, $\lim_{N\to\infty} p(\mathcal{D}|\tilde{\mu}, \tilde{\sigma}^2) \propto \delta(\mu - \tilde{\mu})\,\delta(\sigma^2 - \tilde{\sigma}^2)$. As a result, filter inference with a Gaussian filter is equivalent to ABC based on the mean and variance, $\mathcal{K}_\varepsilon(\mu - \tilde{\mu})\,\mathcal{K}_\varepsilon(\sigma^2 - \tilde{\sigma}^2)$, in the limit $N \to \infty$ and $\varepsilon \to 0$.

Proof: The maximum likelihood estimators (MLEs) of the Gaussian filter likelihood are

$$\mu_* = \frac{1}{N}\sum_{i=1}^N Y_i \quad \text{and} \quad \sigma_*^2 = \sum_{i=1}^N (Y_i - \mu)^2/N,$$

where $\mu_* = \mu$ and $\sigma_*^2 = \frac{N-1}{N}\sigma^2$. The MLEs are the unique maximum of the Gaussian filter likelihood. The curvature of the log-likelihood at the MLEs is

$$\begin{aligned}
\frac{\partial^2}{\partial\tilde{\mu}^2}\log p(\mathcal{D}|\tilde{\mu}, \tilde{\sigma}^2)\Big|_{\tilde{\mu}=\mu_*, \tilde{\sigma}^2=\sigma_*^2} &= -\frac{N}{\sigma_*^2} \\
\frac{\partial^2}{\partial\tilde{\sigma}^2}\log p(\mathcal{D}|\tilde{\mu}, \tilde{\sigma}^2)\Big|_{\tilde{\mu}=\mu_*, \tilde{\sigma}^2=\sigma_*^2} &= \frac{1-N}{\sigma_*^4} \\
\frac{\partial^2}{\partial\tilde{\mu}\,\partial\tilde{\sigma}}\log p(\mathcal{D}|\tilde{\mu}, \tilde{\sigma}^2)\Big|_{\tilde{\mu}=\mu_*, \tilde{\sigma}^2=\sigma_*^2} &= 0.
\end{aligned}$$

In the limit $N \to \infty$, the curvature of the log-likelihood at the MLEs tends to infinity. Due to the monotonicity of the logarithm this implies that the curvature of the likelihood tends to infinity at the MLEs, proving that in the limit $N \to \infty$ a Gaussian filter

likelihood is proportional to a Dirac delta distribution, $\lim_{N\to\infty}\prod_{i=1}^{N} p(\mathcal{D}|\tilde{\mu},\tilde{\sigma}^2) \propto \delta(\mu_* - \tilde{\mu})\,\delta(\sigma_*^2 - \tilde{\sigma}^2)$. In this limit, the maximum likelihood estimator of the variance coincides with the variance estimator, $\lim_{N\to\infty}\sigma_*^2 = \sigma^2$, proving the equivalence between filter inference with a Gaussian filter and ABC based on the mean and the variance in the limit $N \to \infty$ and $\varepsilon \to 0$.
