# Supplementary material for: Filter inference: A scalable nonlinear mixed effects inference approach for snapshot time series data
Source: PLoS Comput Biol. 2023 May 22;19(5):e1011135. doi: 10.1371/journal.pcbi.1011135 (PMC10237648; doi:10.1371/journal.pcbi.1011135)
Supplement: S10 Text — (PDF) [file pcbi.1011135.s010.pdf]

S10 Text. Equivalence of filter inference with identifiable summary statistics-based filters and ABC based on the same summary statistics.

Let $Y_1, \ldots Y_N$ be N i.i.d. random, real-valued variables drawn from the data-generating distribution $q(y)$. Let q have nonzero variance, $\mathrm{Var}[Y] > 0$. Let $\tilde{Y}_1, \ldots \tilde{Y}_S$ be S i.i.d. random, real-valued variables drawn from the model $p(y|\theta)$. Let further $\mathcal{K}_\varepsilon(X - \tilde{X})$ be a kernel with an error margin ε, used in ABC to quantify the distance between the data summary statistic $X = X(Y_1, \ldots Y_N)$ and the simulated summary statistic $\tilde{X} = X(\tilde{Y}_1, \ldots \tilde{Y}_S)$. Let the kernel converge to a Dirac delta distribution up to a proportionality factor as the error margin goes to zero, $\lim_{\varepsilon \to 0} \mathcal{K}_\varepsilon(X - \tilde{X}) \propto \delta(X - \tilde{X})$. Let further $p(y|\tilde{X}_1, \ldots, \tilde{X}_K)$ denote a filter defined by K summary statistics. Let $p(\mathcal{D}|\tilde{X}_1, \ldots, \tilde{X}_K) = \prod_{i=1}^N p(Y_i|\tilde{X}_1, \ldots, \tilde{X}_K)$ denote its likelihood. Let $p(y|\tilde{X}_1, \ldots, \tilde{X}_K)$ be a *summary statistics-based filter*, when the maximum likelihood estimates of the filter likelihood converge to the summary statistics of the data $X_1, \ldots, X_K$ as $N \to \infty$. Let $p(y|\tilde{X}_1, \ldots, \tilde{X}_K)$ be an *identifiable filter*, when the filter likelihood has a unique maximum, whose curvature tends to infinity in the limit $N \to \infty$. Then, an identifiable summary statistics-based filter converges to a Dirac delta distribution between the data summary statistics and the simulated summary statistics up to a proportionality factor as the number of measurements goes to infinity, $\lim_{N \to \infty} p(\mathcal{D}|\tilde{X}_1, \ldots, \tilde{X}_K) \propto \prod_{k=1}^K \delta(X_k - \tilde{X}_k)$. As a result, filter inference with an identifiable summary statistics-based filter is equivalent to ABC based on the same summary statistics, $\prod_{k=1}^K \mathcal{K}_\varepsilon(X_k - \tilde{X}_k)$, in the limit $N \to \infty$ and $\varepsilon \to 0$.

Proof: The MLEs of a summary statistics-based filter recover the summary statistics of the data in the limit $N \to \infty$. The MLEs of an identifiable filter are the unique maximum of the filter likelihood, whose curvature tends to infinity as $N \to \infty$. As a result, the filter likelihood must be proportional to a Dirac delta distribution at the summary statistics of the data in the limit $N \to \infty$, $\lim_{N \to \infty} p(\mathcal{D}|\tilde{X}_1, \ldots, \tilde{X}_K) \propto \prod_{k=1}^K \delta(X_k - \tilde{X}_k)$. The kernel from ABC also converges to a Dirac delta distribution up to a proportionality factor when the error margin goes to zero, $\lim_{\varepsilon \to 0} \prod_{k=1}^K \mathcal{K}_\varepsilon(X_k - \tilde{X}_k) = \prod_{k=1}^K \delta(X_k - \tilde{X}_k)$. As a result, filter inference with an identifiable summary statistics-based filter is equivalent to ABC based on the same summary statistics in the limit $N \to \infty$ and $\varepsilon \to 0$.
