# Supplementary material for: Filter inference: A scalable nonlinear mixed effects inference approach for snapshot time series data
Source: PLoS Comput Biol. 2023 May 22;19(5):e1011135. doi: 10.1371/journal.pcbi.1011135 (PMC10237648; doi:10.1371/journal.pcbi.1011135)
Supplement: S1 Table — (PDF) [file pcbi.1011135.s011.pdf]

S1 Table. Convergence statistics of MCMC chains during the parameter estimation of the early cancer growth model.

	90 IDs (NLME)	90 IDs	270 IDs	810 IDs	2430 IDs
$\hat{R}$ of μ_{y_0}	1.00	1.00	1.00	1.00	1.00
$\hat{R}$ of σ_{y_0}	1.00	1.00	1.00	1.00	1.00
$\hat{R}$ of μ_{λ}	1.00	1.00	1.00	1.00	1.00
$\hat{R}$ of σ_{λ}	1.00	1.00	1.00	1.00	1.00
$\hat{R}$ of μ_{σ}	1.00	1.00	1.00	1.00	1.00
# divergences	0	0	0	0	0
