# Supplementary material for: Filter inference: A scalable nonlinear mixed effects inference approach for snapshot time series data
Source: PLoS Comput Biol. 2023 May 22;19(5):e1011135. doi: 10.1371/journal.pcbi.1011135 (PMC10237648; doi:10.1371/journal.pcbi.1011135)
Supplement: S2 Table — (PDF) [file pcbi.1011135.s012.pdf]

**S2 Table. Convergence statistics of MCMC chains during the parameter estimation of the EGF pathway model.**

| | $\hat{R}$ |
|---|---|
| $\mu_p$ | 1.00 |
| $\sigma_p$ | 1.00 |
| $\mu_{k_{\text{on}}}$ | 1.00 |
| $\sigma_{k_{\text{on}}}$ | 1.00 |
| $\mu_{k_{\text{off}}}$ | 1.00 |
| $\mu_{k_{\text{deg},r}}$ | 1.00 |
| $\mu_{k_{\text{deg},a}}$ | 1.00 |
| # divergences | 0 |
