# Supplementary material for: Filter inference: A scalable nonlinear mixed effects inference approach for snapshot time series data
Source: PLoS Comput Biol. 2023 May 22;19(5):e1011135. doi: 10.1371/journal.pcbi.1011135 (PMC10237648; doi:10.1371/journal.pcbi.1011135)
Supplement: S3 Table — (PDF) [file pcbi.1011135.s013.pdf]

S3 Table. Number of log-posterior evaluations. The table shows the total number of times the log-posterior is evaluated during the inferences in Fig 9.

	90 IDs	270 IDs	810 IDs	2430 IDs	2400 IDs (EGF)
NUTS (warm-up)	20,000	25,626	33,059	53,589	134,413
NUTS (sampling)	14,985	14,985	30,969	30,969	62,937
NUTS (total)	34,985	40,611	64,028	84,558	197,350
Metropolis-Hastings	90,000	90,000	90,000	90,000	180,000
