# Supplementary material for: Filter inference: A scalable nonlinear mixed effects inference approach for snapshot time series data
Source: PLoS Comput Biol. 2023 May 22;19(5):e1011135. doi: 10.1371/journal.pcbi.1011135 (PMC10237648; doi:10.1371/journal.pcbi.1011135)
Supplement: S4 Table — (PDF) [file pcbi.1011135.s014.pdf]

S4 Table. Grid search results: ESS of MH. We ran each MH algorithm with 3 chains for 5000 iterations and computed the ESS after discarding the first 2500 iterations. The reference variances of the filter posteriors are reported in S5 Table and S6 Table.

Scale factor	90 IDs	270 IDs	810 IDs	2430 IDs	2400 (EGF)
1.4	71	**24**	4	3.3	3.14
1.2	71	15	7	3.6	**3.32**
1	**115**	12	**11**	3.3	3.04
0.8	63	16	6	3.2	3.22
0.6	82	7	6	3.6	3.13
0.4	63	8	6	**3.8**	3.08
0.2	52	5	5	3.4	3.29
