# Supplementary material for: Filter inference: A scalable nonlinear mixed effects inference approach for snapshot time series data
Source: PLoS Comput Biol. 2023 May 22;19(5):e1011135. doi: 10.1371/journal.pcbi.1011135 (PMC10237648; doi:10.1371/journal.pcbi.1011135)
Supplement: S5 Table — (PDF) [file pcbi.1011135.s015.pdf]

S5 Table. Variances of filter posteriors: Early cancer model.

	90 IDs	270 IDs	810 IDs	2430 IDs
$\mathrm{Var}[\mu_{y_0}]$	0.086	0.036	0.02	0.017
$\mathrm{Var}[\sigma_{y_0}]$	0.012	0.0084	0.0046	0.0032
$\mathrm{Var}[\mu_{\lambda}]$	0.072	0.036	0.018	0.0014
$\mathrm{Var}[\sigma_{\lambda}]$	0.0082	0.00456	0.0033	0.0024
$\mathrm{Var}[\mu_{\sigma}]$	0.0089	0.1176	0.0082	0.0096
