# Supplementary material for: Filter inference: A scalable nonlinear mixed effects inference approach for snapshot time series data
Source: PLoS Comput Biol. 2023 May 22;19(5):e1011135. doi: 10.1371/journal.pcbi.1011135 (PMC10237648; doi:10.1371/journal.pcbi.1011135)
Supplement: S6 Table — (PDF) [file pcbi.1011135.s016.pdf]

S6 Table. Variances of filter posterior: EGF pathway model.

	Variance
μ_p	0.00005
σ_p	0.0001
$\mu_{k_{\mathrm{on}}}$	0.00006
$\sigma_{k_{\mathrm{on}}}$	0.00004
$\mu_{k_{\mathrm{deg},r}}$	0.0000008
$\mu_{k_{\mathrm{deg},a}}$	0.000002
