# Supplementary material for: Filter inference: A scalable nonlinear mixed effects inference approach for snapshot time series data
Source: PLoS Comput Biol. 2023 May 22;19(5):e1011135. doi: 10.1371/journal.pcbi.1011135 (PMC10237648; doi:10.1371/journal.pcbi.1011135)

S1 Fig. IIV-noise identifiability of early cancer growth model I. The figure shows inference results of the early cancer model using NLME inference and filter inference with a Gaussian filter and $S = 100$ simulated individuals. The parameters are estimated from snapshot datasets with measurements from $N = 90$, $N = 270$, $N = 810$, $N = 2430$ individuals. The measurements are generated for 6 time points between 0 and 0.6 as described in Early cancer growth model inference. The first row shows the posterior distributions inferred from measurements of $N = 90$ individuals. The second, third and fourth row analogously show the results for $N = 270$, $N = 810$ and $N = 2430$. The filter posteriors are illustrated in blue and the NLME posteriors are illustred in red. The prior distributions are illustrated by black dashed lines and the data-generating parameter values are depicted by solid black lines.

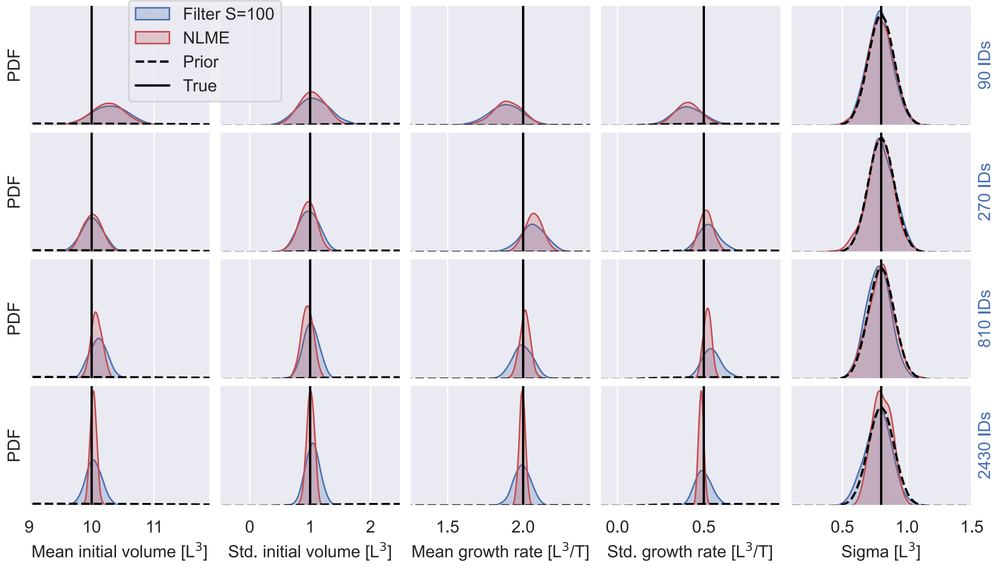

Supplement: S1 Fig — The figure shows inference results of the early cancer model using NLME inference and filter inference with a Gaussian filter and S = 100 simulated individuals. The parameters are estimated from snapshot datasets with measurements from N = 90, N = 270, N = 810, N = 2430 individuals. The measurements are generated for 6 time points between 0 and 0.6 as described in Early cancer growth model inference. The first row shows the posterior distributions inferred from measurements of N = 90 individuals. The second, third and fourth row analogously show the results for N = 270, N = 810 and N = 2430. The filter posteriors are illustrated in blue and the NLME posteriors are illustred in red. The prior distributions are illustrated by black dashed lines and the data-generating parameter values are depicted by solid black lines. (PDF) [file pcbi.1011135.s017.pdf]
