# Supplementary material for: Filter inference: A scalable nonlinear mixed effects inference approach for snapshot time series data
Source: PLoS Comput Biol. 2023 May 22;19(5):e1011135. doi: 10.1371/journal.pcbi.1011135 (PMC10237648; doi:10.1371/journal.pcbi.1011135)

S2 Fig. Early cancer growth measurement distribution. The figure is an extension of S1 Fig and shows the measurement distribution $p(y|\theta, t)$ of the early cancer growth model at $t = 0$, $t = 0.3$ and $t = 0.6$ for three different sets of parameter values. The measurement distribution corresponding to the data-generating parameters, $\theta = (\mu_{y_0}, \sigma_{y_0}, \mu_\lambda, \sigma_\lambda, \mu_\sigma) = (10, 1, 2, 0.5, 0.8)$, is shown in black. A measurement distribution that overestimates the IIV contributions to the measurement variability, $\theta = (10, 1.195, 2, 0.5, 0.47)$, is illustrated in blue, and a measurement distribution that overestimates the noise contributions to the measurement variability, $\theta = (10, 0.7, 2, 0.5, 1.127)$, is illustrated in red.

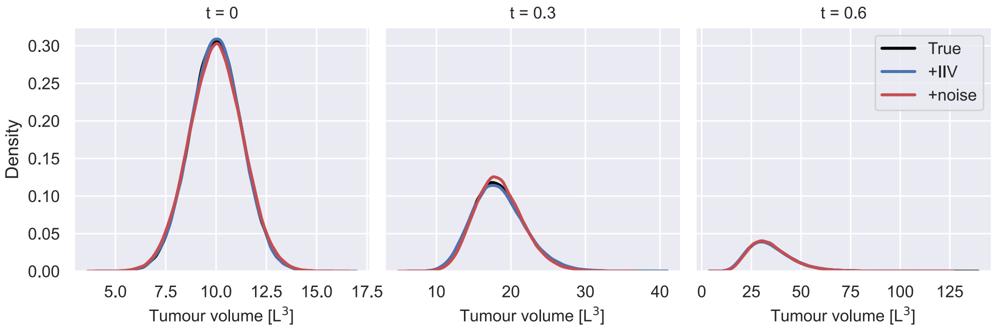

Supplement: S2 Fig — The figure is an extension of S1 Fig and shows the measurement distribution p(y|θ, t) of the early cancer growth model at t = 0, t = 0.3 and t = 0.6 for three different sets of parameter values. The measurement distribution corresponding to the data-generating parameters, θ=(μy0,σy0,μλ,σλ,μσ)=(10,1,2,0.5,0.8), is shown in black. A measurement distribution that overestimates the IIV contributions to the measurement variability, θ = (10, 1.195, 2, 0.5, 0.47), is illustrated in blue, and a measurement distribution that overestimates the noise contributions to the measurement variability, θ = (10, 0.7, 2, 0.5, 1.127), is illustrated in red. (PDF) [file pcbi.1011135.s018.pdf]
