# Supplementary material for: Filter inference: A scalable nonlinear mixed effects inference approach for snapshot time series data
Source: PLoS Comput Biol. 2023 May 22;19(5):e1011135. doi: 10.1371/journal.pcbi.1011135 (PMC10237648; doi:10.1371/journal.pcbi.1011135)

S3 Fig. IIV-noise identifiability of early cancer growth model II. The figure is an extension of S1 Fig and shows filter inference results for the early cancer growth model from 60 000 snapshot measurements using Gaussian filters with $S = 5\,000$ simulated individuals. The prior distributions are illustrated by black dashed lines and the data-generating parameter values are depicted by solid black lines.

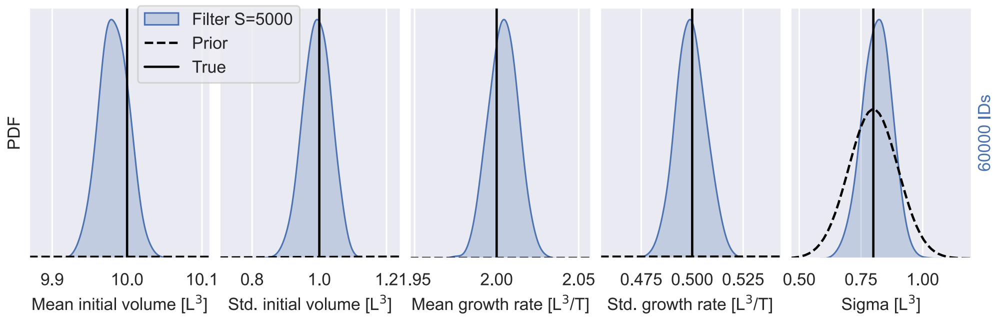

Supplement: S3 Fig — The figure is an extension of S1 Fig and shows filter inference results for the early cancer growth model from 60 000 snapshot measurements using Gaussian filters with S = 5000 simulated individuals. The prior distributions are illustrated by black dashed lines and the data-generating parameter values are depicted by solid black lines. (PDF) [file pcbi.1011135.s019.pdf]
