# Supplementary material for: Filter inference: A scalable nonlinear mixed effects inference approach for snapshot time series data
Source: PLoS Comput Biol. 2023 May 22;19(5):e1011135. doi: 10.1371/journal.pcbi.1011135 (PMC10237648; doi:10.1371/journal.pcbi.1011135)

S4 Fig. Computational costs of log-posterior evaluation with and without gradients. The figure is an extension of Fig 7 and shows the evaluation time of the filter log-posterior with gradients in units of the evaluation time of the filter log-posterior without gradients for different numbers of measured individuals. The evaluation times are estimated according to S7 Text. The left panel shows the results for the early cancer growth model and the right panel the results for the EGF pathway model for $S = 50$ (blue), $S = 100$ (red) and $S = 150$ (green) simulated individuals.

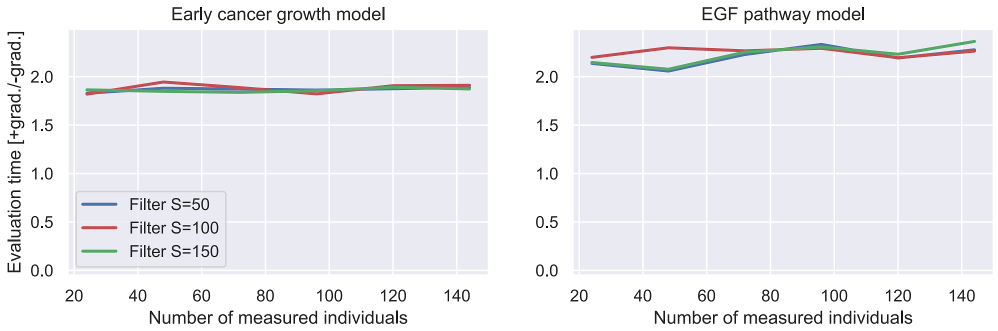

Supplement: S4 Fig — The figure is an extension of Fig 7 and shows the evaluation time of the filter log-posterior with gradients in units of the evaluation time of the filter log-posterior without gradients for different numbers of measured individuals. The evaluation times are estimated according to S7 Text. The left panel shows the results for the early cancer growth model and the right panel the results for the EGF pathway model for S = 50 (blue), S = 100 (red) and S = 150 (green) simulated individuals. (PDF) [file pcbi.1011135.s020.pdf]
